# Interactive Roles of DNA Helicases and Translocases with the Single-Stranded DNA Binding Protein RPA in Nucleic Acid Metabolism

**DOI:** 10.3390/ijms18061233

**Published:** 2017-06-08

**Authors:** Sanket Awate, Robert M. Brosh

**Affiliations:** Laboratory of Molecular Gerontology, National Institute on Aging, National Institutes of Health, NIH Biomedical Research Center, 251 Bayview Blvd, Baltimore, MD 21224, USA; sanket.awate@nih.gov

**Keywords:** helicase, translocase, Replication Protein A, RPA, DNA repair, replication, telomere, checkpoint

## Abstract

Helicases and translocases use the energy of nucleoside triphosphate binding and hydrolysis to unwind/resolve structured nucleic acids or move along a single-stranded or double-stranded polynucleotide chain, respectively. These molecular motors facilitate a variety of transactions including replication, DNA repair, recombination, and transcription. A key partner of eukaryotic DNA helicases/translocases is the single-stranded DNA binding protein Replication Protein A (RPA). Biochemical, genetic, and cell biological assays have demonstrated that RPA interacts with these human molecular motors physically and functionally, and their association is enriched in cells undergoing replication stress. The roles of DNA helicases/translocases are orchestrated with RPA in pathways of nucleic acid metabolism. RPA stimulates helicase-catalyzed DNA unwinding, enlists translocases to sites of action, and modulates their activities in DNA repair, fork remodeling, checkpoint activation, and telomere maintenance. The dynamic interplay between DNA helicases/translocases and RPA is just beginning to be understood at the molecular and cellular levels, and there is still much to be learned, which may inform potential therapeutic strategies.

## 1. Introduction

Helicases are molecular motors that use the energy of nucleoside triphosphate binding and hydrolysis to fuel the separation of the many hydrogen bonds between complementary nucleic acid sequences [1,2,3]. While helicases are often thought of as unwinding duplex nucleic acids (DNA-DNA, DNA-RNA, RNA-RNA), certain specialized helicases can unwind alternatively arranged nucleic acids including DNA triplexes and G-quadruplexes (G4), the latter of which forms in guanine-rich sequences of the genome [4,5,6]. As a class of enzymes, helicases play vital roles in a variety of DNA and RNA transactions that have profound consequences for cellular homeostasis. There has been much progress in the field since the purification and characterization of the first DNA helicase in 1976 [7]. The functions of DNA helicases and specialized molecular motors known as translocases that move along single-stranded or double-stranded DNA are important for DNA repair and maintenance of genomic stability [8,9,10,11]; moreover, an expanding number of genetic disorders are linked to mutations in helicase/translocase genes [12,13,14,15,16,17,18,19,20]. Mutations in genes encoding DNA helicases and translocases are associated with age-related features and cancer [13]. It is evident that their ability to unwind, branch-migrate, or resolve structured mitochondrial and nuclear genomic DNA structures and act in concert with nucleic acid metabolizing enzymes and nucleic acid interacting proteins is paramount to their biological activity.

A key nuclear protein partner of DNA helicases and translocases is Replication Protein A (RPA), a heterotrimeric protein complex consisting of 70 kiloDalton (kD) (RPA70), 32 kD (RPA32), and 14 kD (RPA14) subunits [21]. RPA is an abundant protein, estimated at 30,000–50,000 molecules in human cells [22,23], and 200,000 molecules in transformed human cells [24]. RPA binds to single-stranded DNA with a very high affinity (apparent association constant of 10^9^–10^11^ M^−1^) via its multiple oligonucleotide/oligosaccharide binding (OB) folds, primarily in the RPA70 subunit [25]. Two of the four RPA70 OB-folds constitute the core DNA binding domains. RPA32 and RPA14 each contain 1 OB-fold, and RPA32 contributes by a significantly lesser extent to DNA binding. RPA binding affinity is dependent on single-stranded DNA length. The minimal occluded binding site of human RPA heterotrimer is 8–10 nucleotides (nt), and a more stable 30 nt under physiological conditions. For details on RPA’s structural and biochemical properties, post-translational modifications, and interactions with DNA, see [21,25,26].

Biochemical, genetic, and cellular studies have clearly demonstrated that RPA plays significant roles in replication and the checkpoint response [27], recombination [28,29,30], and DNA repair mediated in part by its protein interactions [31,32]. RPA is an essential protein in eukaryotic cells, and even its partial depletion by RNA interference causes dramatically reduced cell proliferation, decreased DNA synthesis, genomic instability, and poor DNA repair [32,33]. The requirement of RPA for cell viability may help to explain why to date there are no genetic diseases linked to RPA mutations because the single-stranded DNA binding protein is essential for life. However, it remains to be seen if any RPA hypomorphic mutations associated with a reduction in gene expression or partial loss of function are implicated in a disease condition. There is evidence that a missense mutation in the gene encoding RPA70 causes mice to be cancer-prone with the development of lymphoid tumors, accompanied by chromosomal instability and defective double-strand break (DSB) repair [34]. The homologous mutation in human RPA70 causes a defect in single-stranded DNA binding [35], attesting to the physiological importance of its DNA interaction. On the other hand, overexpression of the RPA32 subunit was shown to promote proliferation of breast cancer cells [36]. Other work showed that RPA70 overexpression in lymphoblastoid or glioblastoma cells negatively affected chromosomal stability and DSB repair, leading the authors to speculate that the overabundant RPA may sequester key binding partners [37]; however, a direct investigation of the hypothesis that overexpressed RPA highjacks helicases/translocases or other nuclear factors was not pursued.

The roles of RPA and DNA helicases are intertwined with one another. RPA serves to coat single-stranded DNA and protect it from damage; helicase action generally results in single-stranded DNA formation, which requires RPA’s intervention. Given the importance of RPA in cellular DNA metabolism, we have focused this review on RPA’s interactive roles with human DNA helicases and translocases; however, we include some discussion of helicase-RPA interplay in telomere metabolism and DNA end-resection in yeast, which is likely to lend insight to their collaboration in human cells. We have also addressed the dynamic relationships of RPA with DNA helicases and translocases at the fork during replication stress. Altogether, the collective studies in the field provide strong evidence that helicases/translocases and RPA perform DNA transactions in a concerted manner to maintain cellular and genomic integrity.

## 2. Human RecQ DNA Helicases Interact Physically and Functionally with RPA

Experimental studies from several labs, including ours, have demonstrated that helicases implicated in the replication stress response or DNA repair physically bind to RPA and the single-stranded DNA binding protein stimulates DNA unwinding activity catalyzed by these helicases (Table 1). Among these are the Superfamily (SF) 2 RecQ DNA helicases. From a structural and mechanistic standpoint, the best studied of the helicase-RPA interactions is that of the Werner syndrome (WS) helicase-nuclease (WRN) that is implicated in one of the most striking accelerated aging disorders. Individuals with WS display many clinical features of aging early in life, and typically die by their early 50’s [38]. Although it is well known that cells from WS patients display dysfunctional DNA metabolic characteristics including chromosomal instability, abnormal telomeres, slowed replication fork progression, and hypersensitivity to agents that induce DNA damage or replication stress, it is still unclear what is the exact functional role of WRN in vivo. Characterizing the interactions of WRN with its protein partners may provide important clues to its molecular and cellular functions. Biochemical studies originally from the Loeb lab [39], and then ours [40], showed that RPA dramatically stimulated duplex DNA unwinding by WRN in vitro in a specific manner because heterologous single-stranded DNA binding proteins failed to enhance WRN helicase activity or exerted only a very modest effect. The presence of RPA in reaction mixtures was shown to enable WRN to unwind DNA duplexes up to 850 base pairs (bp) in length, whereas the WRN helicase acting alone was poorly processive and only able to appreciably unwind duplex lengths up to approximately 25–35 bp [40].

The stimulatory effect of RPA on WRN helicase activity was examined from a structural perspective by mapping the protein interaction domains. Using a battery of experimental approaches including yeast two-hybrid mapping, affinity pull-down assays, and enzyme-linked immunosorbent assays (ELISAs) with purified recombinant fragments, it was found that the highly acidic region of WRN residing between the conserved proof-reading nuclease domain in the N-terminal region and the centrally located ATPase/helicase core domain was responsible for the high affinity binding of WRN to a region of RPA70 very close to its basic cleft that also mediates single-stranded DNA binding [41], consistent with RPA70 mapping data from the Loeb lab [42]. In a key set of experiments, it was demonstrated that a purified recombinant full-length WRN protein precisely lacking a strongly acidic domain of perfectly repeated 27 amino acid sequences that is responsible for the high affinity interaction of WRN with RPA70 retained helicase activity on short partial duplex (20 bp) DNA substrates very comparable to the wild-type recombinant WRN helicase, but was very poorly stimulated by RPA to unwind DNA substrates with longer duplexes, even those with only a relatively modestly extended duplex length of 70 bp [41]. Thus, the physical interaction of WRN with RPA as well as RPA’s single-stranded DNA binding ability is required for the functional interaction whereby RPA stimulates WRN helicase activity.

It seems reasonable to assume that the interaction of RPA with WRN and probably other DNA helicases in vivo is most important for DNA metabolic events in which significantly longer stretches of duplex DNA are required to be unwound. Such events would likely include DSB end-resection to establish long single-stranded DNA tracts for homologous recombination (HR) repair (see below), telomere maintenance, or replication fork progression. WRN and RPA may have a specialized role at stalled or blocked replication forks, as suggested by their co-localization in cells treated with the replication inhibitor hydroxyurea (HU) [63], and their enriched association in extracts prepared from cells that have been exposed to DNA damaging agents that would block fork progression [64]. In vitro studies demonstrated that inhibition of WRN helicase activity by bulky benzo[*a*]pyrene adducts on simple forked duplex DNA substrates could be overcome by the presence of RPA in the reaction mixtures [65]. In other biochemical studies, WRN was capable of catalyzing fork regression on forked DNA structures pre-bound with RPA [64], suggesting that WRN and RPA may cooperate with one another at blocked forks. RPA was observed to mediate an indirect interaction of WRN with the DNA translocase SWI/SNF Related, Matrix Associated, Actin Dependent Regulator of Chromatin Subfamily A-Like Protein 1 (SMARCAL1) implicated in fork regression (described below); however, cellular and biochemical data suggest that SMARCAL1 and WRN operate independently of one another at stalled forks [66]. RPA was demonstrated to be capable of stimulating WRN branch-migration activity on plasmid-based displacement (D)-loop DNA substrates in vitro, and this activity may be relevant to WRN’s involvement in HR or telomere metabolism when such structures arise [43].

**Figure 1:**
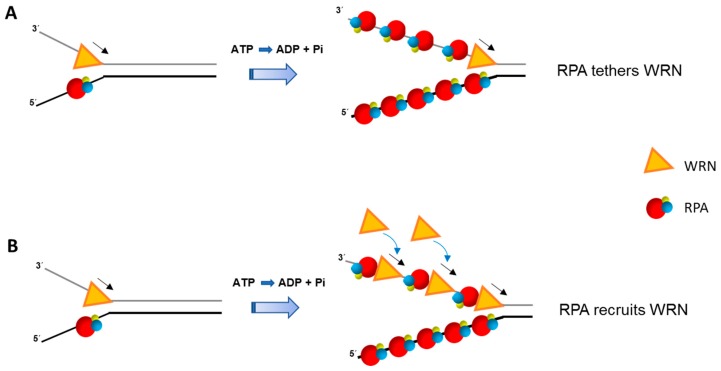
Replication Protein A (RPA) stimulates double-stranded DNA unwinding by the Werner syndrome (WS) helicase-nuclease (WRN) helicase in a manner that is still poorly understood. Two potential mechanisms whereby the physical interaction of RPA with WRN facilitates unwinding of relatively long duplex DNA tracts are depicted. RPA bound to the unwound single-stranded DNA keeps the WRN molecule tethered at the single-stranded/double-stranded DNA junction so that it continues to catalyze unwinding in a processive manner (**A**). Alternatively, RPA helps to recruit additional WRN helicase molecules in solution to facilitate duplex unwinding (**B**). The two models are not mutually exclusive and may depend on the DNA structural intermediate or cellular state. Note that positional placement of WRN relative to RPA in (**A**) and (**B**) is arbitrary. Adenosine triphosphate (ATP); Adenosine diphosphate (ADP); inorganic phosphate (Pi). RPA heterotrimer is represented by spheres of red (RPA70), blue (RPA32), and yellow (RPA14). Small black arrow indicates directionality of WRN helicase translocation.

The mechanism for RPA stimulation of WRN helicase activity even on simple duplex DNA substrates remains to be fully understood. Presumably, RPA enhances WRN-catalyzed DNA unwinding by coating the unwound single-strands and interacting with WRN helicase to keep it tethered at the single-stranded/double-stranded junction during unwinding of relatively long duplex DNA tracts. Alternatively, RPA coated onto single-stranded DNA at the fork junction may help to recruit additional WRN molecules from solution to aid duplex unwinding (Figure 1).

Single-turnover and single-molecule experiments may prove to be highly informative for deciphering if RPA makes WRN more processive or aids in its recruitment to the advancing fork. Interestingly, presteady state kinetic analysis demonstrated that WRN (in the absence of RPA) unwound a 20 bp forked duplex DNA substrate as a monomer [67], raising the question if RPA might tether WRN monomer to the DNA substrate for the helicase to unwind longer DNA duplexes or if it alters WRN’s assembly state to make it more processive. The 5′ to 3′ single-stranded DNA binding polarity of RPA and its multiple DNA binding modes [68,69,70] have prompted some discussion of how RPA might coordinate its interactions with DNA substrate intermediates during DNA processing events (for review, see [25]). Fanning et al. proposed that RPA may ‘unroll’ during binding to single-stranded DNA or RPA may dissociate in a sequential manner, in which case RPA’s modular nature would provide a dynamic element for the interaction of other proteins (e.g., DNA polymerases, helicases) with the DNA substrate [71]. Clearly, further work is required to understand the molecular mechanism(s) involved in RPA stimulation of the 3′ to 5′ eukaryotic RecQ helicases or 5′ to 3′ helicases like human Fanconi Anemia Group J (FANCJ) (discussed below), which may be distinct from one another.

It is likely that mechanisms exist in the cell to modulate the WRN-RPA interaction so that helicase-catalyzed DNA unwinding can be regulated. The tumor suppressor p53 interacts with both RPA [72,73] and WRN [74,75], and biochemical results demonstrate that p53 inhibits RPA-dependent and RPA-independent WRN helicase activity [76]. p53-mediated apoptosis is attenuated in WS cells [75], raising the possibility that the interaction of p53 with WRN or the WRN-RPA complex provides a signal during replication for programmed cell death to remove cells from the population that are unable to repair DNA damage. p53 is also thought to regulate HR via its interaction with WRN or the sequence-related Bloom’s syndrome helicase (BLM) [77] (discussed below), and it is conceivable that the post-translational phosphorylation of p53 and/or RPA by checkpoint kinases which facilitates their dissociation [78] allows WRN or BLM to promote HR repair.

RPA physically and functionally interacts with the human RecQ helicase BLM that is mutated in Bloom’s syndrome (BS) [44], a growth retardation disorder characterized by elevated sister chromatid exchange and a strong incidence of cancer [79]. RPA also interacts with ATP-dependent DNA helicase Q-like 1 (RECQL1) [46,47], which is important for chromosomal stability [80] but not yet genetically linked to a hereditary disorder; nonetheless, RECQL1 mutations are associated with breast cancer [81,82,83]. For both BLM and RECQL1, the stimulation of helicase-catalyzed DNA unwinding was specific as evidenced by the poor ability of heterologous single-stranded binding proteins to affect helicase activity. Consistent with this, both BLM and RECQL1 physically interact with RPA via the RPA70 subunit, suggesting a conserved interaction with that of WRN. In fact, mapping studies demonstrated that an N-terminal acidic region of BLM, like WRN, is engaged in the physical interaction with RPA70 [41]. Despite the evidence for a strong biochemical interaction of BLM or RECQL1 with RPA, the biological significance of these interactions remains poorly understood. The BLM-RPA interaction may be important for double Holliday Junction (HJ) dissolution to suppress crossovers during HR, an activity dependent on BLM as first shown by the Hickson lab [84]. The double HJ dissolution reaction mediated by the BLM-Topoisomerase (Topo) IIIα-RMI1-RMI2 complex has been further characterized biochemically [85,86]. The Sung lab determined that RPA physically interacts with an acidic region of RMI1, and this interaction is required for efficient double HJ dissolution [45]. It remains to be determined how RPA juggles its interactions with RMI1 and BLM in the BLM-Topo IIIα-RMI1-RMI2 complex, and if the physical interaction of RPA with BLM is also required for double HJ dissolution.

RECQL4 is genetically implicated in the rare disorders Rothmund-Thomson syndrome, RAPADILINO syndrome, and Baller-Gerold syndrome [87]. The weak DNA unwinding catalyzed by purified recombinant RECQL4 was only modestly stimulated by RPA on a 22 bp forked duplex DNA substrate; furthermore, no stimulation was observed for a 30 bp forked duplex [88]. In contrast, DNA unwinding of a 30 bp forked duplex substrate by human RECQL5β (the largest of three isoforms) was strongly stimulated by RPA; moreover, RPA stimulated RECQL5β branch-migration of a synthetic HJ substrate [49]. Whether RECQL5β’ s catalytic activity on a key HR intermediate is important in vivo remains to be formally proven; however, Recql5-deficient mice are cancer-prone and human RECQL5β can displace the major strand recombinase protein Rad51 in an ATP hydrolysis and RPA-dependent manner in vitro [50], suggesting potentially multiple levels of HR regulation. Although neither RECQL5β nor RECQL4 were reported to physically bind RPA, the differential effects of RPA on their respective catalytic activities warrants further investigation.

All five human RecQ helicases have been shown to perform annealing of complementary DNA strands in vitro, and the effect of RPA on their respective strand annealing activities has been assessed in each case. RECQL5β strand annealing was first reported to be inhibited when the single-stranded DNA was pre-coated by RPA [49], suggesting that the helicase is not able to effectively displace RPA bound to the single strands. Strand annealing by RECQL1 [89], BLM [90], WRN [91], and RECQL4 [92] are also inhibited by RPA in vitro. It is tempting to speculate that strand annealing by the human RecQ helicases may be important for a pathway of HR repair such as synthesis-dependent strand annealing in which successive cycles of DNA unwinding and strand annealing followed by DNA synthesis take place to rejoin the broken DNA ends; however, the observation that RPA inhibits strand annealing activity by the human RecQ helicases distinguishes them from BRCA2, which can promote Rad51 filament formation on RPA-coated single-stranded DNA [93]. Perhaps strand annealing by human RecQ helicases may play a role when the single-strand tracts are sufficiently short (<~10 nucleotides) to preclude stable binding by RPA [25], in which case alternative end-joining mediated by microhomology of ≤10 bp is sufficient [94]. Alternatively, it is conceivable that strand annealing by RecQ helicases may occur when RPA is exhausted under conditions of replication stress ([95] and next section). Nonetheless, to our knowledge there is yet no biological significance attributed to strand annealing catalyzed by any of the human RecQ helicases.

## 3. RECQL1 Governs RPA during Replication Stress

In addition to the direct interaction of RPA with human RecQ helicases, we recently discovered a dynamic interplay between human RECQL1 status and the free RPA pool during replication stress. RECQL1 ensures that the rate of DNA synthesis by elongating replication forks remains reasonably fast even during replication stress so that there is not an over-abundance of dormant origin firing which would titrate free RPA away from the forks already established [96]. By doing so, nascent single-stranded DNA generated at the fork is coated by RPA to protect it from damage. Thus, RECQL1 plays a critical role in allowing RPA to be available to ensure normal replication fork dynamics, suppress DNA damage, and preserve genomic stability. Our findings are reminiscent of the earlier observations by Toledo et al. [95] that the ataxia telangiectasia and Rad3-related (ATR) checkpoint kinase helps to avert replication catastrophe by also preventing global RPA exhaustion. RECQL1’s ability to direct an RPA-dependent response to stressed forks in cancer cells by ensuring a fast replication rate and suppressing dormant origin firing suggests that the helicase may pose an ideal target to combat cancer. Although WRN-specific helicase inhibitors [97,98,99] and a BLM helicase inhibitor [100] have been identified and used in cell-based studies, there are yet no reports of a RECQL1 small molecule inhibitor. It will be of interest to determine if pharmacological inhibition of DNA unwinding by RECQL1 (or the other human RecQ helicases) in cancer cells disrupts RPA dynamics during replication stress. The topic of small molecule inhibitors is further addressed in the Concluding Remarks.

## 4. Replication Fork Remodeling by the DNA Translocase SMARCAL1 Is Controlled by Its Interacting Partner RPA

DNA damage, nucleotide starvation, unusual DNA structures, and transcription complexes are among the obstacles that can perturb normal DNA synthesis during cellular replication [101]. One mechanism that cells use to deal with stalled replication forks is to regress the fork into a four-way junction, the so-called “chicken foot” structure. By doing so, the DNA lesion presented in duplex DNA can be bypassed by reverse branch-migration and fork restart and then the lesion repaired later, or the lesion can be immediately repaired because it is exposed in the double-strand state by the act of fork regression. Fork regression (or fork remodeling as it is also called) is important for the suppression of replication-associated DSBs. A key player in fork remodeling is a specialized DNA translocase known as SMARCAL1 (also designated HepA-related protein (HARP)) which is genetically linked to Schimke immune-osseous dysplasia, a disease with multiple defects involving the kidney, immune system, and cancer [102]. Purified recombinant SMARCAL1 protein was originally characterized to possess ATP-dependent annealing activity on complementary single-stranded DNA sequences bound by RPA [103]. In very recent work using a single-molecule magnetic tweezers assay, Burnham et al. obtained experimental data indicating that SMARCAL1-catalyzed ATP hydrolysis reanneals hundreds of bp by step-wise removal of RPA in a non-processive manner, thereby allowing rehybridization of the strands in a process that is likely dependent on multiple SMARCAL1 molecules [104].

**Figure 2:**
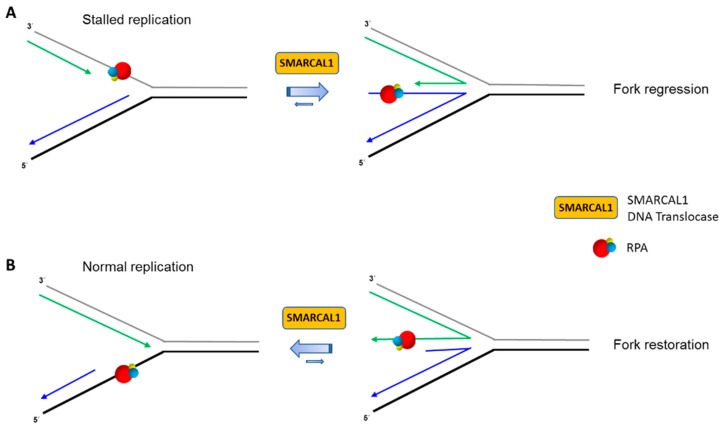
RPA regulates SMARCAL1 function at replication forks. RPA binding to single-stranded DNA at replication fork structures with defined polarity modulates ATP-dependent SMARCAL1 DNA translocase in a manner that dictates the fate of stalled (**A**) or normal (**B**) replication forks. RPA heterotrimer is represented by spheres of red (RPA70), blue (RPA32), and yellow (RPA14). Physical interaction of RPA32 with SMARCAL1 is not shown. Nascent leading and lagging strands are indicated by green and blue arrows, respectively. See reference [59] and text for details.

SMARCAL1 physically binds to the RPA32 subunit, and RPA helps to guide SMARCAL1 to stalled forks [105,106,107,108,109,110]. The interaction of SMARCAL1 with RPA32 is distinct from the RecQ helicases WRN [41], BLM [44], and RECQL1 [46], as well as the Fe-S helicase FANCJ [51] (see below), which all interact with the RPA70 subunit. Biochemically, RPA stimulates SMARCAL1 to catalyze fork regression [58,66]. In an elegant set of experiments with engineered recombinant SMARCAL1 mutant proteins and DNA substrates with defined polarity sub-structural DNA elements, the Cortez lab teased out how RPA with its high affinity single-stranded DNA binding domains confers SMARCAL1 substrate specificity in an orientation-dependent manner mediated by the single-stranded DNA binding protein’s polarity of interaction with the DNA substrate [59]. Furthermore, domain swapping experiments demonstrated that a SMARCAL1 mutant protein in which the RPA32 interaction motif was substituted with a RPA70 interaction motif was regulated by RPA in a similar manner to that of the wild-type recombinant SMARCAL1 in the in vitro fork regression reactions. Thus, high affinity RPA binding to the DNA fork in a polarity-specific manner is the dominating factor for SMARCAL1’s stimulation of regression on a modeled stalled fork or inhibition of regression on a modeled normal fork (Figure 2).

Presumably, one of RPA32’s primary roles is to aid in the recruitment of SMARCAL1 to the stalled fork in vivo, which would not be expected for the human RecQ or FANCJ helicases shown to physically bind RPA70, not RPA32 (Table 1). Nonetheless, it remains to be seen how RPA subunits orchestrate with other DNA translocases and helicases on biologically relevant DNA structures. However, it should be noted that a recombinant protein representing the N-terminal half of RPA70, which is able to bind single-stranded DNA or WRN, is capable of stimulating WRN helicase activity on a simple partial duplex DNA substrate [42]. There may be fundamental differences between RPA stimulation of DNA unwinding by a helicase versus DNA fork remodeling by a translocase. In the case of SMARCAL1, the evidence from the Cortez lab suggests that RPA heterotrimer binding to the leading strand template stimulates SMARCAL1-catalyzed fork regression when the fork is stalled. On the other hand, RPA heterotrimer binding to the nascently synthesized leading strand of a regressed fork stimulates SMARCAL1-catalyzed fork remodeling in such a manner as to restore the fork (Figure 2). Understanding the molecular mechanics of how RPA facilitates the directed action of DNA molecular motors like SMARCAL1 will surely yield new insights into the still poorly understood events associated with fork remodeling and other DNA transactions.

## 5. Functional Interactions of Fe–S Cluster Helicases with RPA

Members of the SF 2 Iron–Sulfur (Fe–S) cluster family, including Xeroderma pigmentosum Group D (XPD, DEAD/H Box 11 (DDX11) (also called Chromosome loss-related 1(ChlR1)), Regulator of telomere elongation helicase 1 (RTEL1), and FANCJ, play prominent roles in DNA repair and maintenance of genomic stability [20]. Genetic defects in all four of these Fe–S helicases result in diseases with distinct features. The first to be characterized was XPD, which is genetically implicated in Xeroderma pigmentosum, Cockayne’s syndrome, Trichothiodystrophy, and Cerebro-oculo-facio-skeletal (COFS) syndrome [111]. As a member of the general transcription factor TFIIH, XPD is implicated in both transcription and nucleotide excision repair (NER) responsible for the removal of bulky DNA lesions. Evidence suggests that RPA positions the nucleases critical for the removal of the oligonucleotide containing the damaged base [69,112]. Although human XPD is not reported to physically or functionally interact with RPA, it seems reasonable to postulate their coordination in the steps of damage verification and/or excision, but this requires further investigation. Limited expansion of the NER or transcription bubble is a possibility.

Unlike the human RPA heterotrimer, two forms of *F. acidarmanus* RPA (*Fac*RPA) exist: (1) *Fac*RPA1 homodimer with 2 OB-folds; (2) *Fac*RPA2 monomer with 1 OB-fold. The Spies lab tested for a functional interaction of these *Fac*RPA proteins with *Fac*XPD and found that *Fac*RPA2 stimulated *Fac*XPD helicase activity significantly better than *Fac*RPA1 [55]. Among the findings reported in this study, three stand out: (1) *Fac*RPA2 acting alone melted a fraction of the forked duplex (20 bp) DNA substrate; (2) In the presence of *Fac*RPA2, *Fac*XPD unwound the 20 bp forked duplex as a monomer; (3) By DNA footprint analysis, it was inferred that *Fac*RPA2 melted duplex, allowing advancement of *Fac*XPD helicase. Based on their results, the authors suggested the requirement of either duplex destabilization or trapping of unwound strands (or both) by *Fac*RPA2 for stimulation of *Fac*XPD helicase activity. Human RPA heterotrimer is also known to destabilize duplex DNA [113,114], raising the question if it operates at the single-stranded/double-stranded DNA junction to melt duplex and facilitate helicase-catalyzed DNA unwinding. As alluded to in Figure 1, RPA may help to tether a single helicase molecule to the DNA substrate or recruit helicase molecules from solution during unwinding while also serving to coat the unwound strands emerging from the translocating helicase. It is of interest to determine if the sequence-related FANCJ helicase, which interacts physically and functionally with RPA [51], unwinds duplex DNA as a monomer or as a dimer which is its more optimal catalytic state in the absence of RPA [115]. Clearly, more sophisticated studies including single molecule analysis will be required to address these possibilities.

Recessive bi-allelic mutations in the Fe-S helicase DDX11/ChlR1 are linked to the rare genetic disorder Warsaw Breakage syndrome characterized by microcephaly and congenital abnormalities, as well as defective sister chromatid cohesion at the cellular level [116,117]. ChlR1 interacts with several DNA replication proteins, including the Okazaki fragment processing enzyme FEN-1 [54]. It is thought that ChlR1 protein interactions and helicase activity enable the cell to efficiently process DNA replication intermediates to allow cohesin deposition [118]. Acting alone, ChlR1 could unwind a partial duplex DNA substrate of 100 bp, and to a much lesser extent longer duplexes; however, in the presence of RPA, ChlR1 unwound partial duplex substrates up to 500 bp in length [54]. A reconstituted system to study the process of sister chromatid cohesion and the factors involved will be required to characterize the importance of RPA-stimulated ChlR1 helicase activity and ChlR1 protein interactions in cohesin loading, which is still poorly understood in terms of mechanics.

The most recent Fe-S helicase to be implicated in human disease is RTEL1, in which recessive bi-allelic mutations are linked to a severe form of Dyskeratosis congenita known as Hoyeraal-Hreidarsson syndrome (HHS) [119]. Cells from HHS patients are known to display telomere fragility, and it is believed that RTEL1 unwinds specialized three-stranded DNA structures (D-loops) that arise from HR strand invasion or exist as specialized telomeric (T)-loops at chromosome ends [120,121]. Although to our knowledge an interaction of RPA with RTEL1 has not been reported, their putative coordination would be relevant to the finding that extracts from human cells expressing ATPase-active (but not ATPase-inactive) RTEL1 disrupt a plasmid/oligonucleotide based T-loop substrate in vitro [121]. Given that the artificial T-loop substrate used in that study has an invading duplex region of ~50 bp, it seems probable that longer T-loop duplexes likely to exist in vivo would require ancillary factors like RPA for efficient RTEL1-catalyzed resolution.

## 6. FANCJ-RPA Interaction, Replication Stress, and Checkpoint Activation

FANCJ, originally designated BRCA1-interacting C-terminal helicase 1 (BACH1) (or BRCA1-interacting protein 1 (BRIP1)) for its interaction with the tumor suppressor BRCA1 [122], is genetically implicated in the progressive bone marrow failure disorder Fanconi Anemia (FA) [123,124,125]. In addition to anemia, individuals with FA often present with congenital abnormalities and a strong predisposition to cancer; moreover, mono-allelic FANCJ mutations are associated with breast cancer [123]. FANCJ along with currently 20 other genes implicated in FA, constitute a pathway for the repair of DNA interstrand cross-links (ICL) [126]. FANCJ is believed to play a role in the downstream step(s) of the pathway to repair processed cross-links or DSBs by HR [127]. In addition to its roles in DNA repair, FANCJ was found to associate with telomeres that are maintained by the alternative lengthening of telomeres (ALT) pathway [128,129]. The first regulatory protein partner of FANCJ identified was RPA [51]. Like the RecQ helicases, FANCJ physically binds to the RPA70 subunit and the RPA heterotrimer stimulates duplex DNA unwinding by FANCJ in a specific manner [51]. Furthermore, RPA co-localizes with FANCJ in human cells that have been exposed to agents that induce DNA damage (ionizing radiation, mitomycin C) or stall replication fork progression (HU), and this enriched association is dependent on BRCA1 [51].

**Figure 3:**
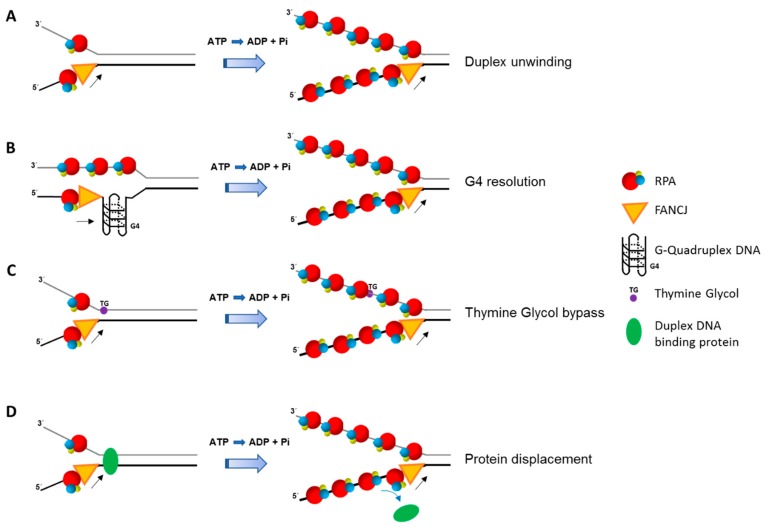
RPA enhances multiple ATP-dependent activities catalyzed by FANCJ. RPA stimulates FANCJ unwinding of duplex DNA (**A**) or resolving G-quadruplex DNA (**B**). RPA also enhances FANCJ’s ability to bypass a non-translocating strand thymine glycol (**C**) or displace protein bound to duplex DNA (**D**). Adenosine triphosphate (ATP); Adenosine diphosphate (ADP); inorganic phosphate (Pi). RPA heterotrimer is represented by spheres of red (RPA70), blue (RPA32), and yellow (RPA14). Small black arrow indicates directionality of FANCJ helicase translocation. Small blue arrow indicates displacement of duplex DNA binding protein. See text for details.

RPA affects FANCJ activity in additional ways other than simply enhancing its helicase activity on simple duplex DNA substrates (Figure 3). RPA stimulates FANCJ resolution of G4 DNA substrates [52], and enables FANCJ to unwind past a thymine glycol residing in the strand opposite to the one the helicase translocates [53]. The strand-specific stimulation of FANCJ helicase activity past thymine glycol involves the high affinity binding of RPA to the exposed thymine glycol in the single-stranded state. Like FANCJ, RECQL1 helicase activity on a DNA substrate harboring a thymine glycol is stimulated by RPA in a strand-specific manner, suggesting a conserved mechanism for lesion bypass even though the two helicases have opposite polarities of unwinding [53]. RPA also strongly enhances FANCJ’s ability to displace protein bound to duplex DNA [48]. Therefore, FANCJ and RPA are likely to collaborate in multiple aspects of DNA metabolism, including efficient replication past sites of DNA damage or protein obstacles. FANCJ’s requirement for a 5′ single-stranded tail in order to unwind the adjacent G4 structure, and the demonstration that RPA stimulates FANCJ unwinding of the G4 substrate with a 5′ single-stranded DNA tail [52], suggests their interactive role during DNA synthesis in which the unreplicated 5′ single-stranded end of the G4 becomes bound by RPA which stimulates FANCJ resolution of the G4 [130].

Cell-based studies have provided a clue to how FANCJ might be involved with RPA to deal with replication stress via an intracellular signaling mechanism. Checkpoint activation is a critical event that allows cells to cope with DNA damage or pharmacologically induced replication stress by halting the progression of the cell cycle, stabilize forks, promote DNA repair, and modulate transcription [131,132,133]. TopBP1 is a non-enzymatic scaffold protein that activates ATR, which is responsible for G2/M checkpoint activation in response to replication stress [134]. ATR activation occurs when there is uncoupling between replicative helicase DNA unwinding at the fork and DNA synthesis by the polymerase which is stalled. The resulting single-stranded DNA is bound by RPA which serves to recruit TopBP1 and activate ATR. Gong et al. detected a direct interaction between TopBP1 and FANCJ that is mediated by S-phase specific phosphorylation of FANCJ [135]. Depletion of FANCJ, or TopBP1, curtailed RPA loading onto chromatin and ATR-dependent phosphorylation of Chk1 that is normally induced by replication stress. A model was proposed in which the TopBP1-FANCJ interaction is required for single-stranded DNA extension and RPA coating the single-stranded DNA at stalled forks, which in turn is required for the activation of the replication checkpoint. While this model is attractive, it remains to be understood how these events are coordinated with the myriad of other factors (e.g., 9–1–1, RFC) that are recruited to stalled forks and known to be involved in checkpoint activation, and if the stimulation of FANCJ helicase activity by RPA is required for single-strand production at the stalled fork. Post-translational modification of FANCJ (or RPA) by phosphorylation may modulate their physical/functional interaction, and influence the creation of single-stranded DNA at the stalled fork, which would in turn potentially affect ATR-mediated checkpoint activation.

## 7. Involvement of DNA Helicases with RPA in Double-Strand Break End-Resection

DSBs in replicating eukaryotic cells are repaired with greatest fidelity by HR using complementary sequence of the sister chromatid in a complex process characterized by sequential steps. Much insight into the players involved was first gleaned from yeast studies. Long-range resection is mediated by two independent pathways: one catalyzed by DNA replication ATP-dependent helicase/nuclease (Dna2) and the RecQ helicase Sgs1 and the other by Exonuclease 1 (Exo1). Exo1 has an intrinsic 5′ to 3′ exonuclease activity and can degrade the double-stranded DNA end with the appropriate polarity by itself to generate the 3′ single-stranded tail in vitro that is required for HR to occur [136]. Studies from the Seo lab showed that in the absence of RPA, Dna2 preferentially degrades DNA with a 5′ single-stranded tail, but is also active on DNA with a 3′ single-stranded tail [137]. In subsequent work, RPA was found to sequester the single-stranded DNA unwound by Sgs1 and provide directionality to Dna2 nuclease by inhibiting its 3′ to 5' degradation activity [138,139]. Therefore, long-range resection catalyzed by the Sgs1-Dna2 complex requires the presence of RPA [136]. Sgs1 unwinds DNA in a 3′ to 5′ direction and the unwound single-stranded DNA with the free 5′ end is digested by the helicase-nuclease Dna2.

The findings described above are consistent with work which showed that a yeast Dna2 mutant defective in ATP hydrolysis retains its ability to function in DNA end-resection [138,139]. However, very recent studies demonstrated that in an *exo1* mutant strain, Dna2 ATPase activity is important for resection [140,141]. Dna2-catalyzed ATP hydrolysis enables the 5′ to 3′ movement of Dna2 along the single-stranded DNA flap until it reaches the single-strand: double-stranded junction where Dna2 catalyzes incision. RPA’s interaction with Dna2 regulates cleavage polarity and is required for the efficient 5′ to 3′ motor activity of Dna2. These findings suggest a revised model for Dna2 mediated resection in which Sgs1 first unwinds double-stranded DNA near the end trimmed by the Mre11-Rad50-Xrs1 (MRX)-single-stranded DNA endodeoxyribonuclease (Sae2) (homologs of human Mre11-Rad50-Nbs1 (MRN)-crossover hotspot initiator protein (CtIP)) complex. RPA’s interaction with Sgs1 stimulates processive unwinding of the double-stranded DNA. ATP hydrolysis by Dna2 enables its 5′ to 3′ movement along the flap until it reaches the junction where it creates an incision. Cleaved ssDNA is further processed by Dna2. RPA prevents 3′ to 5′ movement of Dna2 on the single-stranded DNA flap. Thus, RPA interactions with Sgs1, Dna2 and the 5′ single-stranded DNA flap are critical to the process of long range DNA end-resection.

Chen et al. conducted an elegant study in yeast to examine the in vivo role of RPA in DNA end-resection [142]. They used a heat-inducible degron system to conditionally deplete Rfa1 (yeast homolog of human RPA70) and found that RPA depletion had no effect on the initial resection mediated by the MRX-Sae2 complex. However, the long-range end-resection carried out by both the Sgs1-Dna2 and Exo1 pathways were determined to be dependent on RPA, consistent with the observation that depletion of RPA resulted in the reduced formation of Rad51 foci. By binding to the 3′ single-stranded DNA overhang produced by initial resection, RPA prevented short repeat sequences from forming hairpin structures to allow helicase/nuclease-dependent strand resection.

**Figure 4:**
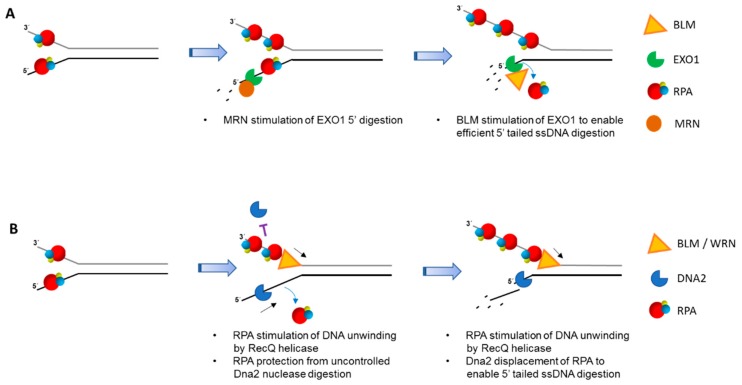
RPA-interacting helicases and DNA nucleases involved in double-strand break (DSB) long range resection generate 3′ single-stranded tails for homologous recombination (HR) repair. After initial trimming by the MRN-CtIPcomplex (not shown), RPA binds single-stranded DNA near ends, thereby preventing inappropriate nucleolytic DNA degradation. (**A**), MRN, collaborating with RPA, stimulates 5′ to 3′ end-resection by EXO1. Further EXO1 5′ to 3′ end-resection is enhanced by Bloom’s syndrome helicase (BLM) protein interaction with EXO1 that is independent of BLM helicase activity. In the process, RPA becomes unbound from the degraded end. (**B**), RPA is poised to stimulate DNA unwinding by BLM and/or WRN helicases, creating longer single-stranded DNA. In addition, RPA protects single-stranded DNA with a free 3′ end from being degraded by Dna2 nuclease activity. The 5′ to 3′ helicase-nuclease DNA2 is directed by RPA binding to degrade 5′-ended single-stranded DNA. In both (**A**,**B**), the long 3′ single-stranded DNA tails are used for RAD51-mediated strand pairing with complementary DNA strand of recipient duplex for HR repair (not shown). RPA heterotrimer is represented by spheres of red (RPA70), blue (RPA32), and yellow (RPA14). Small black arrow indicates directionality of BLM/WRN helicase translocation.

Biochemical reconstitution of DNA end-resection with purified recombinant human proteins demonstrated that, like yeast, two pathways exist involving BLM-DNA2-RPA-MRN and EXO1-BLM-RPA-MRN [143]. In the EXO1 pathway, RPA enhances EXO1’s affinity for DNA ends. MRN stimulated EXO1 resection even in the presence of RPA, whereas BLM enhanced further 5′ single-stranded tail digestion by EXO1 (Figure 4A). In the DNA2 pathway, RPA serves to not only stimulate BLM helicase activity, but also confer the 5′ to 3′ resection polarity by DNA2 (Figure 4B). Genetic and biochemical evidence has more recently indicated that human WRN can substitute for BLM in the DNA2/RPA-dependent resection pathway [144].

Tammaro et al. used Xenopus egg extracts to study the role of RPA in end-resection and showed that an RPA70 N-terminal deletion mutant that is unable to interact with WRN or Dna2 failed to stimulate WRN helicase or Dna2 helicase/nuclease activities, respectively [145]. The RPA70 N-terminal deletion mutant retained its ability to bind single-stranded DNA, suggesting that RPA’s physical interaction with WRN and Dna2 is critical for the end-resection process. Thus, RPA serves a dual role in WRN-Dna2 mediated resection: (1) RPA interacts with the single-stranded DNA to prevent formation of the hairpin structures and keeps the unwound strands separated; (2) RPA’s physical interaction with WRN and Dna2 stimulates WRN helicase and Dna2 exonuclease activities. The RPA heterotrimer binds to the single-stranded DNA in such a way that the N-terminal domain of RPA70 is able to interact with WRN/BLM or Dna2 positioned at the 5′ end of the ssDNA it is bound. This spatial positioning of the RPA70 N terminal domain is optimal for the recruitment of WRN to the single-stranded/double-stranded DNA junction and movement of the helicase in a 3′ to 5′ direction to unwind the duplex DNA. The RPA70 N-terminal domain also recruits Dna2 in an optimal manner, wherein it binds and displaces RPA from the 5′ ssDNA overhang, stimulating the end-resection in a 5′ to 3′ direction.

Resection is a key step that must occur in the 5′ to 3′ direction for HR repair to occur. Initial resection by MRN complex serves as a platform for the recruitment of RPA which then interacts and recruits Dna2. The ability of Dna2 to act on the RPA-coated single-stranded DNA depends on the physical interaction between RPA and Dna2. Zhou et al characterized the crystal structure of full-length mouse DNA2 with the OB-fold domain of human RPA70 bound to single-stranded DNA [146]. DNA2 was found to be cylindrical in shape with the nucleolytic active sites embedded in the center of the tunnel. The size of the tunnel was determined to be too narrow to allow the passage of double-stranded DNA, or single-stranded DNA bound by RPA. DNA2 was observed to bind and displace RPA from a 5′ single-stranded DNA overhang, but was unable to displace it from a 3′ overhang. The results suggested that displacement of RPA results in free 5′-ended single-stranded DNA which threads itself into the DNA2 tunnel where the active site of the nuclease domain is located. This allows DNA2-mediated resection to occur only in the 5′ to 3′ direction [146].

## 8. Interaction and Interplay between HELB (HDHB) and RPA

Research from the Enomoto lab [147,148] and the lab of the late Ellen Fanning [56,149,150] elucidated an important role of mammalian DNA helicase B (HELB, also referred to as human DNA helicase B (HDHB) in human cells), in chromosomal DNA replication. Replication stress in human cells was shown to cause HELB to recruit to chromatin in an RPA-dependent manner [56]. An acidic region in HELB physically interacts with RPA via an N-terminal basic cleft of the RPA70 subunit that is also important for recruiting S phase checkpoint signaling proteins to chromatin. Although a functional interaction of RPA to stimulate DNA unwinding by HELB was not reported, transient depletion of HELB compromised cellular recovery from pharmacologically induced replication stress and elevated chromosomal instability, attesting to the importance of HELB in vivo.

The physical interaction sites of HELB and RPA70 for each other are reminiscent of the WRN acidic domain interaction with a basic cleft of RPA [39,41,42]. Because all the known human RPA-interacting DNA helicases bind to RPA70 (Table 1), it is tempting to speculate that an ionic interaction between the basic cleft of RPA70 and an acidic domain of the helicase represents a conserved interaction mode. In support of this idea, an N-terminal region of the BLM protein which contains acidic regions was found to harbor a single high affinity interaction site for RPA essential for stimulation of BLM helicase activity on an 850 bp partial duplex DNA substrate [41]. Mapping studies with the other RPA70-interacting helicases may provide further evidence for a conserved interaction mode. If so, genetic complementation assays with site-directed helicase mutants defective in the RPA70 interaction may help to tease out the delegation of tasks by the RPA-interacting helicases.

More recent studies have provided further insight into the interplay between HELB and RPA. Depletion of RPA in human cells was observed to impair HR and cause delayed late-stage RPA foci formation after ionizing radiation exposure, suggesting that RPA fails to efficiently bind displaced single-stranded DNA generated by Rad51-mediated strand invasion into recipient duplex [149]. Consistent with this, HELB was shown to stimulate Rad51-mediated 5′ to 3′ heteroduplex extension in vitro. The authors proposed a model in which HELB helicase activity possibly operating in concert with the double-stranded DNA translocase Rad54 expands the displacement loop, thereby allowing RPA to coat the displaced strand during heteroduplex extension.

Interplay between HELB and RPA was discovered at yet another level when researchers were screening for proteins that regulate the RPA-dependent end-resection step of HR-mediated DSB repair [57]. HELB was confirmed to interact with RPA, which facilitates its recruitment to sites of DSBs. In an RPA-dependent manner, ATPase-active HELB acts to inhibit end-resection catalyzed by EXO1 or BLM-DNA2, suggesting a mechanism to regulate HR during the cell cycle and avoid chromosomal instability. The observation that HELB-depleted BRCA1-deficient tumor cells were resistant to a PARP inhibitor is consistent with an auto-regulatory mechanism to limit end-resection dependent on HELB and its interaction with RPA [57]. This finding may have implications for PARP-targeted anti-cancer strategies which rely on compromised DSB repair in BRCA1-deficient tumors.

## 9. Pfh1 and Its Interplay with RPA to Lengthen Telomeres and Preserve Their Stability

There is ample evidence for a critical role of eukaryotic SF 1 Pif1 family helicase members (*S. cerevisiae* Pif1, *S. pombe* Pfh1) in telomere maintenance [151]. RPA stimulates Pif1-catalyzed unwinding of partial duplex DNA substrates, and to a lesser extent DNA-RNA substrates [60], but the significance of their functional interaction in vivo is unclear. Recent advances implicate an interactive role of Pfh1 helicase with RPA to facilitate telomere lengthening in *S. pombe*. McDonald et al. carefully examined the Pfh1-RPA interaction in *S. pombe* using a series of experimental approaches including two-dimensional gel electrophoreses and analyses of Pif1 protein and DNA interactions at the telomeres by co-immunoprecipitation, mass spectrometry, and chromatin immunoprecipitation-Sequence (ChIP-Seq) identification [61]. Exogenously expressed Myc-tagged Pfh1 displayed increased association with telomeres compared to other genomic sites, consistent with a specialized role of the helicase at chromosome ends. Moreover, the exogenously expressed Pfh1 was shown to be a positive regulator of telomere length in a manner that was dependent on its intrinsic ATPase/helicase activity but not on the DNA recombinase Rad51, suggesting that Pfh1’s telomeric role is not dependent on HR.

Surprisingly, overexpression of Myc-tagged Pfh1 did not increase the association of the telomere-interacting factors Pot1, Trt1, or Est1 with telomeres, suggesting another mechanism whereby Pfh1 enables telomere lengthening [61]. It was previously shown that both *S. pombe* RPA and *S. cerevisiae* RPA associate with telomerase via its direct interaction with the telomerase RNA template (TLC1) [152], leading McDonald et al. [61] to explore if RPA’s interaction with telomeric single-stranded DNA might be necessary for Pfh1 to lengthen telomeres in vivo. To address this hypothesis, the researchers evaluated the effect of myc-Pfh1 expression in a *S. pombe* strain designated *rad11-D223Y* which harbors a missense mutation in the OB-fold of the largest RPA subunit (RPA70) that is important for high affinity single-stranded DNA binding. The *rad11-D223Y* strain was already known to have shorter telomeres than those of the wild-type yeast strain. Myc-Pfh1 overexpression in the *rad11-D223Y* strain failed to increase the length of the short telomeres [61]. This result, taken together with the observations that RPA was observed to interact with Pfh1 [62] and Myc-Pfh1 overexpression increased RPA binding to telomeres [61], suggest that Pfh1 increased the interaction of RPA with telomerase, which was responsible for the telomere lengthening [61].

Recently, the impact of the Rpa1-D223Y mutation on *S. pombe* telomere length was examined independently by Audry et al. [153]. Using an expression system in which Pfh1 was overexpressed from a very strong promoter (*nmt1*), they observed that the short telomere length was rescued in the *rad11-D223Y* strain. Consistent with the idea that overexpressed Pfh1 resolved telomeric G4 structures to maintain telomere length, purified recombinant *S. pombe* Pfh1 was found to resolve G4 DNA structures in vitro [154]. In addition to *S. pombe* Pfh1, other G4 unwinding helicases, including the *S. cerevisiae* Pif1, could rescue the short telomere phenotype of the *S. pombe rad11-D223Y* strain. This led Audry et al. to suspect that secondary DNA structure that accumulates during replication in the G-rich sequence of the lagging strand due to a RPA deficiency caused telomere defects that could be suppressed by expression of a G4-resolving helicase [153]. Biochemical studies demonstrated that the D223Y mutation in human RPA significantly diminished its G-quadruplex binding ability [153]. Rad52 was recruited to the telomeres in *rpa-1D223Y* cells, suggesting that HR may help to resolve the secondary structures that accumulate [153], but this idea was not experimentally addressed further in the study. Nonetheless, the defective recruitment of shelterin proteins to telomeres in *rad11-D223Y* mutant cells [153], together with the other findings, suggested that RPA may serve to diminish G4 formation at lagging strand telomeres to allow shelter binding and telomerase extension of the telomere. Consistent with this idea, several groups have shown that RPA binds to G4 DNA structures, including those derived from human telomeric sequence, and destabilize them [155,156,157,158,159]. As an additional line of defense, the eukaryotic family of Pif1 helicases serve to suppress G4-associated genomic instability at telomeres and other regions of the nuclear genome (for review, see [151]).

## 10. Collaboration among Helicases, RPA, and Shelterin Proteins to Remove Secondary DNA Structure at Telomeres and Facilitate Their Replication or Repair

The destabilization of telomeric G4 and sequestration of single-stranded DNA by RPA may aid shelterin proteins to load helicases onto chromosome ends to facilitate DNA repair or replication of telomeres. Zimmerman et al. proposed that the shelterin protein TRF1, which binds to the double-stranded hexanucleotide repeat, recruits BLM to telomeres to enable efficient lagging strand telomere DNA synthesis [160]. Thus RPA, shelterin proteins, and helicases like BLM or WRN [161] may operate collaboratively to remove secondary structure and preserve telomere stability thereby ensuring efficient replication or repair of telomeric DNA. Given the strong evidence from the Karlseder group that cells from WS patients are defective in replicating the telomeric G-rich lagging strand template [162,163], and WRN is found at telomeres where it interacts with members of the shelterin complex [164], it is probable that WRN performs the timely resolution of G4 DNA and removal of T-loops via its coordinate helicase and exonuclease activities to ensure telomere stability in vivo. Consistent with a G4-resolving role, deficiency in either human RecQ helicase led to the greatest abundance of G4 structures at telomeres [165]. It is plausible that human PIF1 helicase, which has opposite polarity to that of WRN and BLM, plays a role in the metabolism of telomeric G4 DNA structures, given that it can also resolve G4 DNA [166], and its overexpression rescued the telomere defects of the *rad11-D223Y* strain [153]. Although it is highly probable that Pif1 helicase activity is engaged in the resolution of unusual DNA structures at telomeres, the importance of a putative interaction between human PIF1 and RPA in telomere metabolism has not been elucidated.

Because unfolded G4 DNA can readily snap back into its G4 topology, we favor the idea that RPA, shelterin proteins, and G4 resolving helicases all collaborate with one another to allow the G-rich telomeric sequences to assume a single-stranded state at least transiently so that they can be copied by the replisome, elongated by telomerase, or repaired by relevant DNA repair machinery. For example, telomeres are considered sinks for oxidative DNA damage which would be anticipated to be corrected by the base excision repair machinery [167,168,169,170]. Dynamic interactions and interplay of helicases with single-stranded DNA binding proteins like RPA or POT1, as well as involvement of shelterin proteins that bind telomeric duplex DNA (e.g., TRF1, TRF2) are likely to be important for helicases to maintain their length, thereby promoting cellular homeostasis. Interestingly, the SMARCAL1 DNA translocase is implicated in telomere maintenance [171,172], but this function is apparently independent of RPA binding by SMARCAL1 [172].

## 11. Concluding Remarks

Biochemical and cellular studies have provided strong evidence that eukaryotic DNA helicases coordinate their activities with RPA in the context of DNA repair, replication stress response, fork remodeling, checkpoint activation, and telomere metabolism. However, mechanistic aspects detailing the direct interactions of RPA with DNA helicases, and their interplay in a biological context remain to be further characterized. It is very apparent that the physical and functional interactions of RPA with the human RecQ helicases and certain Fe-S cluster helicases (e.g., FANCJ) are paramount to their biochemical and cellular functions. Certain new or underappreciated aspects of RPA function, including RPA sliding on single-stranded DNA [173], as well as the ability of helicases to displace or bypass RPA bound to single-stranded DNA [174], suggests new avenues of helicase research for both in vitro and in vivo studies. The dynamic interactions of helicases or helicase-like proteins with DNA structures harboring single-stranded elements coated by RPA at stalled replication forks is certainly of great interest, as illustrated by the studies of the disease relevant DNA translocase SMARCAL1.

**Figure 5:**
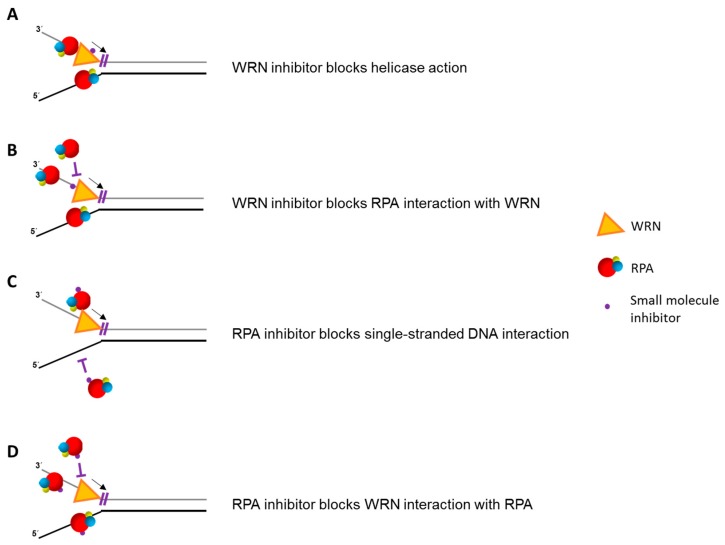
Small molecule drugs that target helicase-RPA interactions. Biologically active small molecules that bind to a DNA helicase, thereby inhibiting its unwinding function directly (**A**) or disrupting its physical interaction with RPA (**B**), may be used to target helicase-dependent pathways of DNA metabolism in human cells. Alternatively, RPA-interacting compounds that interfere with single-stranded DNA binding (**C**) or physical interaction with a helicase (**D**) may compromise helicase-dependent pathways. RPA heterotrimer is represented by spheres of red (RPA70), blue (RPA32), and yellow (RPA14). Small black arrow indicates directionality of WRN helicase translocation. Small purple T symbol represents inhibition of designated interaction or action of WRN or RPA. Note that small molecule inhibition mechanisms are not necessarily mutually exclusive.

From a pre-clinical and translational viewpoint, it is of interest to explore if targeting the interaction and interplay of RPA with human DNA helicases might pose some therapeutic benefits. Helicase inhibitors are already being studied as a prospective target for anti-cancer therapy [97,175,176], and there is also interest in RPA-interacting small molecules to enhance chemotherapeutic strategies as well [177,178,179,180,181]. Given the closely intertwined roles of helicases with RPA in the DNA damage and replication stress response, it will be informative to explore if small molecules can modulate their interaction and cross-talk in a manner that might be suitable for development in the clinic (Figure 5).

In conclusion, DNA helicases and translocases play vital roles in nucleic acid metabolism that are frequently orchestrated with RPA. RPA not only greatly enhances helicase-catalyzed DNA unwinding, but also recruits DNA translocases to sites of action such as the replication fork during periods of DNA damage and DNA polymerase stalling. By its strong single-stranded DNA binding and protein interactions, RPA modulates the functions of DNA helicases in DNA repair, fork remodeling, checkpoint activation, and telomere maintenance. Thus, therapeutic strategies may be on the horizon that target the dynamic relationships of RPA with cellular DNA helicases.

## Figures and Tables

**Table 1:** Physical and functional interactions of DNA helicases and translocases with RPA.

Helicase	SF ^1^/Family	Physical Interaction	Functional Interaction with RPA Heterotrimer	Reference
WRN	SF2 RecQ	RPA70; acidic region	Stimulates WRN dsDNA ^2^ unwinding	[39,40,41,42]
			Stimulates WRN D-loop branch migration	[43]
BLM	SF2 RecQ	RPA70	Stimulates BLM dsDNA unwinding	[41,44]
			Stimulates BLM-Topo3α double HJ dissolution	[45]
RECQL1	SF2 RecQ	RPA70	Stimulates RECQL1 dsDNA unwinding	[46,47]
			Stimulates RECQL1 protein-DNA displacement	[48]
RECQL5β	SF2 RecQ	ND ^3^	Stimulates RECQL5β dsDNA unwinding	[49]
			Stimulates RECQL5β displacement of Rad51	[50]
FANCJ	SF2 Fe–S	RPA70	Stimulates FANCJ dsDNA unwinding	[51]
			Stimulates FANCJ G4 DNA resolution	[52]
			Stimulates FANCJ bypass of thymine glycol	[53]
			Stimulates FANCJ protein-DNA displacement	[48]
DDX11	SF2 Fe–S	ND	Stimulates DDX11 dsDNA unwinding	[54]
FacXPD	SF2 Fe–S	ND	FacRPA2 ^4^ stimulates FacXPD dsDNA unwinding	[55]
HELB	SF1 RecD	RPA70	ND	[56,57]
SMARCAL1 ^5^	SNF2 SWI/SNF	RPA32	Stimulates SMARCAL1 fork remodeling	[58,59]
ScPif1 ^6^	SF1 Pif1	ND	Stimulates Pif1 dsDNA and RNA-DNA unwinding	[60]
SpPfh1 ^7^	SF1 Pif1	RPA70/32/14	ND	[61,62]

^1^ SF, Superfamily; ^2^ dsDNA, double-stranded DNA; ^3^ ND, not determined; ^4^ FacRPA2 tested; ^5^ DNA translocase; ^6^ Sc, S. cerevisiae; ^7^ Sp, S. pombe.
